# Factors related to the performance of laypersons diagnosing pigmented skin cancer: an explorative study

**DOI:** 10.1038/s41598-023-50152-x

**Published:** 2023-12-21

**Authors:** Nadja Beeler, Esther Ziegler, Alexander A. Navarini, Manu Kapur

**Affiliations:** 1 Professorship for Learning Sciences and Higher Education, ETH Zurich, Clausiusstrasse 59, 8092 Zurich, Switzerland; 2 Department of Dermatology, University Hospital Basel, Burgfelderstrasse 101, 4055 Basel, Switzerland

**Keywords:** Melanoma, Melanoma

## Abstract

It is important but challenging for prospective health professionals to learn the visual distinction between potentially harmful and harmless skin lesions, such as malignant melanomas and benign nevi. Knowledge about factors related to diagnostic performance is sparse but a prerequisite for designing and evaluating evidence-based educational interventions. Hence, this study explored how the characteristics of 240 skin lesions, the number of classified lesions and the response times of 137 laypeople were related to performance in diagnosing pigmented skin cancer. Our results showed large differences between the lesions, as some were classified correctly by more than 90% and others by less than 10% of the participants. A *t*-test showed that for melanomas, the correct diagnosis was provided significantly more often than for nevi. Furthermore, we found a significant Pearson correlation between the number of solved tasks and performance in the first 50 diagnostic tasks. Finally, *t*-tests for investigating the response times revealed that compared to true decisions, participants spent longer on false-negative but not on false-positive decisions. These results provide novel knowledge about performance-related factors that can be useful when designing diagnostic tests and learning interventions for melanoma detection.

## Introduction

The distinction between malignant—potentially harmful—and benign—harmless—pigmented skin lesions is a key skill of future dermatologists and, to some extent, even general physicians^1^. One frequent type of skin lesions are melanocytic lesions, such as malignant melanomas (also known as pigmented skin cancer) and benign nevi. Unfortunately, the visual distinction between melanomas and nevi is challenging to learn because they share many common visual features^2^. However, false-negative diagnoses are life-threatening, as melanoma is the most lethal form of skin cancer and accounts for more than 80% of all skin cancer deaths^3^. At the same time, false-positive diagnoses lead to needless surgery and scarring. Therefore, detecting melanoma reliably is an essential skill for prospective health professionals. Still, there is empirical evidence that graduates often do not meet the recommendations for competence to detect skin cancer^4^. Hence, there is an urgent need to improve current training methods. Unfortunately, finding evidence on how to design effective instruction for visual learning in medicine in general and melanoma detection specifically is difficult^5^. Therefore, studies should aim to develop and compare learning interventions for melanoma detection and explore the underlying learning mechanisms to explain a method's potential superiority. Like this, research can contribute to a better understanding of how visual learning works, which can be used to improve learning interventions^6^.

### The importance of performance-related factors

To conduct research that allows in-depth insights into visual learning in skin lesion classification, it is crucial to know about factors related to the diagnostic performance in visual tasks, such as characteristics of the lesions, number of solved classification tasks and response time. However, learning studies for skin lesion classification with human subjects have thus far usually neither provided a detailed description of their image datasets nor explored performance-related factors in detail^2,7–9^. Contrastingly, our recently published work showed how knowledge about these performance factors can inform and improve study designs in two ways^10,11^: First, it is a prerequisite for designing interventions that allow fair comparisons between different learning treatments, for example, by balancing relevant lesion characteristics influencing the probability of a correct diagnosis. Second, understanding these factors also allows for a more differentiated interpretation of the results, for example, stratified analyses by task difficulty. With this explorative study, we provide the empirical foundation for such sophisticated study designs by investigating three factors that are potentially related to performance in melanoma detection: (1) characteristics of the skin lesions, (2) the number of solved tasks, and (3) response time, which we describe in the following sections.

(1) *Characteristics of the skin lesions*

The first factor that might be associated with diagnostic performance is the characteristics of the skin lesions. The present study explores potential performance differences between 240 image classification tasks, as well as the impact of the five characteristics diagnosis, size, and anatomic site of the lesions, plus the age and biological sex of the patients.

(2) *Number of solved tasks*

The second factor potentially related to performance in visual diagnostic tasks is the number of solved tasks. It would be useful to know to what extent the diagnostic performance of individuals who have to solve multiple visual tasks can be expected to increase due to adaptation^12^ and testing effects^13,14^ or to decrease due to fatigue^15^. Hence, the present study investigates how laypersons’ performance changes during the sequence of 80 diagnostic tasks being solved.

(3) Response time

The third factor that could be related to performance is the response time^16^. Previous research has mainly shown a negative relationship between response time and performance in diagnostic tasks, meaning that more time is spent on false than true decisions^17–19^. However, the response times for false-positive, false-negative, true-positive and true-negative decisions have not been investigated in detail, although potential differences might be important to consider when using response time as an indicator for (meta-)cognitive processes^16,20^. Therefore, this study explores the relationship between response time and diagnostic performance in depth.

## Methods

### Participants

We recruited 154 participants from Amazon Mechanical Turk. Inclusion criteria were US citizens, age ≥ 18 years, approval rate ≥ 98% and the number of solved human intelligence tasks ≥ 50. The participants provided informed consent, and they received 1.5$ for completing the survey and 0.1$ per correctly solved task. The ethics committee of ETH Zurich approved the study (EK 2020-N-160). All methods were performed in accordance with the Swiss Federal Act on research involving human beings and the ordinance on human research, with the exception of clinical trials.

The data of 17 participants were removed before the analysis either because of the time they needed to answer the survey or because their decision behaviour showed anomalies (Appendix A). The highest level of education of the remaining 137 participants was mainly a Bachelor’s degree (56.2%), followed by a Master’s degree (18.2%), high school graduates (16.8%), and associate’s degrees (8.8%). Most of the participants said they had no (41.6%), little (23.4%), or some (19.0%) experience with skin lesions; 16.1% said they had a lot of experience. 22.6% indicated no skin lesions on their body, 43.1% reported having more than zero but less than 20 lesions, 31.4% between 20 and 100, and 2.2% more than 100 lesions.

### Surveys

We selected dermoscopic images of biopsy-proven 120 malignant melanomas and 120 benign nevi from the International Skin Imaging Collaboration archive^21^ according to pre-defined inclusion criteria (Appendix B) to achieve a standardised dataset. Subsequently, we randomly distributed the selected skin lesions to three online surveys so that each survey finally included images of 40 melanomas and 40 nevi. Post-randomisation checks were well-balanced across the surveys (Appendix C).

**Figure 1:**
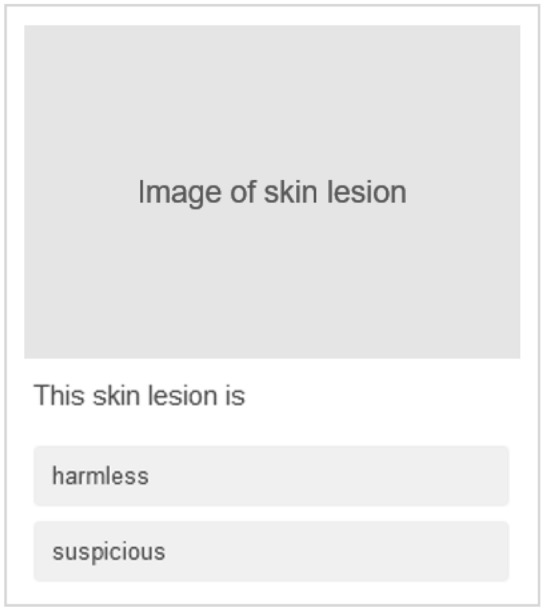
Illustration of a skin lesion classification task.

Using the selected images, we designed skin lesion classification tasks in which the participants were shown one image per page of the survey and indicated the presumed correct classification in single-choice questions (Fig. 1). Each image of a skin lesion corresponded to one classification task. We decided to provide the answer options *harmless* or *suspicious* rather than *nevus* or *melanoma* because we investigated laypersons without prior knowledge and wanted them to focus on the classification task rather than thinking about technical terms. The participants were randomly assigned to the three surveys, and the sequence of tasks was randomised for each participant. Between 45 and 47 subjects classified each of the 240 lesions, resulting in 10,960 decisions that were analysed.

There were no time limits for classifying the individual or all images. The median time for classifying all 80 images per survey (without reading the instructions and answering the final demographic questions) was 5.17 min (*SD* = 3.09 min, *Min* = 2.13 min, *Max* = 15.42 min).

### Analyses

Table 1 provides an overview of the analysis procedures. Details are provided in the following sections.

**Table 1:** Overview of executed analysis procedures. The aim of the statistical analyses was always to evaluate how the three investigated factors (1–3) were related to the performance indicators (A, B).

Performance-related factor	Analysis perspective	
	Tasks	Individuals
	**Performance indicator = ** **A) Task performance** = **Proportion of the participants who suggested the correct diagnosis**	**Performance indicator = ** **B) Individual performance** = **Accuracy = (TP + TN)/(FP + FN)**
**(1) Characteristics of the skin lesions**	- Diagnosis (task performance in melanomas vs. nevi): Two-sided *t*-test for independent samples - Clinical size of the lesion: Pearson correlation between size and task performance - Age of the patient: Pearson correlation between age and task performance - Anatomic site of the lesion: One-way analysis of variance for task performance of different sites - Sex of the patient (task performance in lesions from female vs. male patients): Two-sided *t*-test for independent samples	Not applicable
**(2) Number of solved tasks**	- Plot of moving average of task performance - Pearson correlations between the number of solved tasks and task performance	Not applicable
**(3) Response time**	*- Inter-tasks perspective:* Pearson correlation between task response time and task performance *- Intra-tasks perspective:* One-sided paired samples *t*-tests (Hypothesis: Task response time for incorrectly solved tasks > for correctly solved tasks)	*- Inter-individuals perspective:* Pearson correlation between individual response time and accuracy *- Intra-individuals perspective:* One-sided paired samples *t*-tests (Hypothesis: Individual response time for false decisions > for true decisions)

#### Analysis perspectives

We performed analyses from two different perspectives. On the one hand, we ran analyses from a *tasks perspective* to investigate whether the properties of the tasks were related to the performance in these tasks. On the other hand, we analysed the data from an *individuals perspective* to investigate whether the behaviour of the individuals was decisive for their performance. We analysed all three potential performance-related factors (lesion characteristics, number of solved tasks, and response time) from a *tasks perspective*. For the response time, we additionally performed analyses from an *individuals perspective* (Table 1). Details on the analysis methods for both perspectives are provided in the following sections. We checked for all performance indicators and performance-related factors that the data were approximately normally distributed with Kolmogorov–Smirnov tests and by visual inspection of histograms.

#### Performance indicators

**Figure 2:**
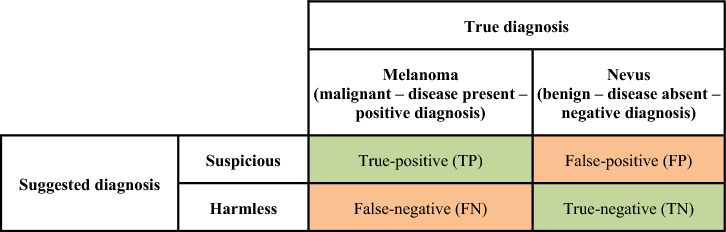
Confusion matrix.

From a *tasks perspective*, we used the proportion of the participants who suggested the correct diagnosis in each task (the so-called *task performance)* as a performance indicator. To analyse the performance from an *individuals perspective*, we first assessed for each of the participants’ decisions, whether they were true-positive (TP), true-negative (TN), false-positive (FP) or false-negative (FN), as displayed in the confusion matrix in Fig. 2. Subsequently, we calculated accuracy ((TP + TN)/(FP + FN)) to assess the *individual performance* of each person. We omitted to calculate further performance measures, such as F1-Score, because we used a balanced dataset. In this case, accuracy provides the most comprehensive performance indicator (i.e., true-positives and true-negatives have equal weight).

#### Performance-related factors

(1) Characteristics of the skin lesions

To investigate how the characteristics of the skin lesions were related to the task performance, we first conducted a two-sided Student’s *t*-test for independent samples to compare the task performances between melanomas versus nevi. Subsequently, we analysed the other skin lesion characteristics differentiated for the two categories of lesions because it is plausible that the relationship between the task performance and these characteristics differs between melanomas and nevi. For the lesion's clinical size and the patient's age (continuous variables), we calculated Pearson correlations between the respective variable and the task performance. For the anatomic site of the lesion (categorical variable), we calculated a one-way analysis of variance. For the sex of the patient (binary variable), we conducted a two-sided Student’s *t*-test for independent samples to assess potential differences in task performance.

(2) Number of solved tasks

We plotted the moving average of the task performance throughout all 80 tasks per participant to visually inspect a potential relationship with the number of solved tasks. Furthermore, we calculated Pearson correlations between the number of solved tasks and task performance.

(3) Response time

The *raw response time* corresponded to the time from the moment the task was displayed until the participants submitted their suggested diagnosis. From a *tasks perspective*, we subsequently assessed the *task (response) time* by calculating the median based on the raw response times of multiple participants per task. From an *individuals perspective*, we evaluated the *individual (response) time* by calculating the median based on the raw response times of multiple tasks per participant. For reader-friendliness, we will call these *task time* and *individual time* from now on.

For both analysis perspectives, we found the median better suited to assess the central tendency for each task/individual than the average because the time data was right-skewed for most tasks/individuals. However, for further analyses, we calculated the average time for multiple tasks/individuals (based on the median time per task/participant), as this data was approximately normally distributed.

From an *inter-tasks perspective*, we calculated Pearson correlations between task time and task performance. In addition, from an *intra-tasks-perspective*, we performed one-sided paired samples *t*-tests to test the hypothesis that task times are longer for incorrectly than for correctly solved tasks. From an *inter-individuals perspective*, we calculated Pearson correlations between individual time and individual performance (accuracy). Furthermore, from an *intra-individuals perspective*, we conducted one-tailed *t*-tests for dependent samples to test the hypotheses that individual times are longer for false than for true decisions in each of the following pairs:

1. All false (FP & FN) versus all true (TP & TN) decisions
2. False versus true decisions in melanoma tasks (FN vs. TP)
3. False versus true decisions in nevus tasks (FP vs. TN)
4. False versus true decisions when suggesting the diagnosis *suspicious* (FP vs. TP)
5. False versus true decisions when suggesting the diagnosis *harmless* (FN vs. TN)

## Results

 (1) Characteristics of the skin lesions

**Figure 3:**
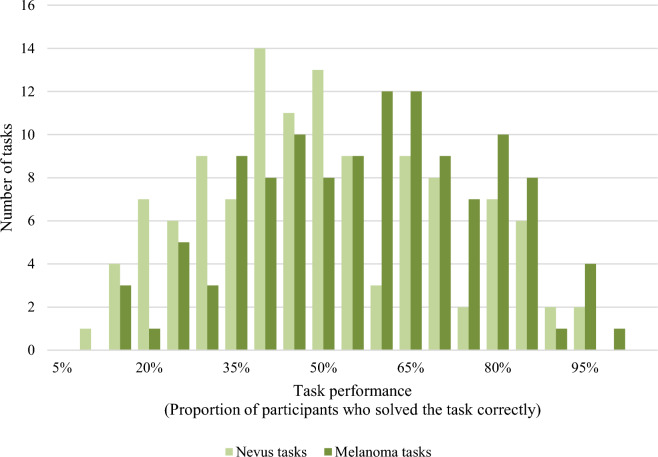
Histogram of task performances.

We found large differences in the task performances: *M* = 51.3%, *SD* = 20.7%, *Min* = 8.89%, *Max* = 95.7%, as illustrated in Fig. 3. Furthermore, a *t*-test for independent samples showed that the average task performance was significantly higher for tasks in which melanomas (*M* = 55.2%, *SD* = 20.2%), compared to nevi (*M* = 47.4%, *SD* = 20.5%) had to be classified (*t*(238) = 2.982, *p* = 0.002, Cohen’s *d* = 0.385).

For the melanoma tasks, the Pearson correlations between the task performance and the clinical size of the lesion (*r* = − 0.047, *p* = 0.610), as well as the age of the patient (*r* = − 0.039, *p* = 0.669), were not significant. The result of the one-way ANOVA indicated no significant differences in the task performances between the three anatomic sites torso, extremities and head/neck (*F*(2, 117) = 1.892, *p* = 0.155, *η*^*2*^ = 0.031). Lastly, the independent samples *t*-test did also not reveal any significant differences in the average task performance between melanomas stemming from female (*M* = 57.7%, *SD* = 19.9%) versus male (*M* = 53.4%, *SD* = 20.3%) patients (*t*(118) = 1.164, *p* = 0.247, Cohen’s *d* = 0.215).

For the nevus tasks, we again found no significant correlations between the task performance and the clinical size of the lesion (*r* = − 0.167, *p* = 0.68) and the patients’ age (*r* = − 0.056, *p* = 0.540). The one-way-ANOVA to compare potential differences in the task performance between the three anatomic sites was also non-significant (*F*(2, 117) = 2.732, *p* = 0.070, *η*^*2*^ = 0.044). Finally, the Student’s *t*-test for independent samples revealed that the task performance was significantly higher in nevi stemming from female (*M* = 50.9%, *SD* = 20.2%) versus male (*M* = 43.3%, *SD* = 20.3%) patients (*t*(118) = 2.056, *p* = 0.042, Cohen’s *d* = 0.377). Some additional analyses to explore the characteristics of the skin lesions in-depth are described in Appendix D.

(2) Number of solved tasks

**Figure 4:**
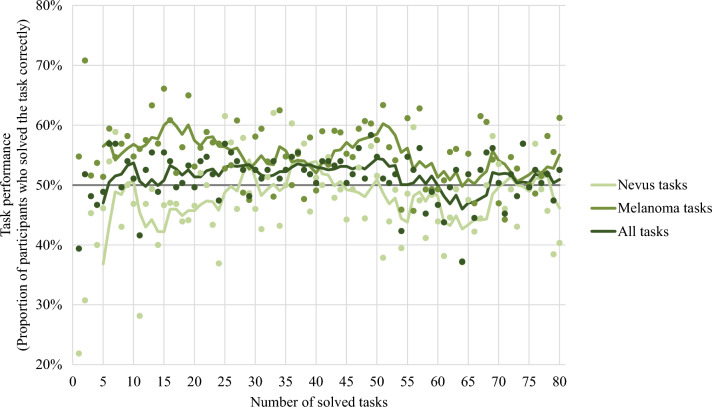
Development of task performance over the number of solved tasks. *Legend:* Dots represent the task performance in single tasks; lines represent the moving average of the task performance over five tasks. An average task performance of 50% could be expected by chance (grey line).

The plot of the task performance over the number of solved tasks (Fig. 4) shows that the overall task performance increases slightly, or at least stayed stable, for the first 50 tasks. Then there was a decrease until approximately task 65, when the task performance started to increase again until it reached a comparable level as in the first tasks, approximately from task 70 to 80. The plot furthermore indicates that the slight increase in task performance in the first 50 tasks was primarily due to an increase in nevus tasks. In contrast, the performance in melanoma tasks stayed relatively stable. The decrease in performance starting from task 50 was reflected in a decreasing performance in both the melanoma and the nevus tasks.

For all 80 tasks per survey, the Pearson correlation between the number of solved tasks and the overall task performance (*r* = − 0.066 *p* = 0.559) was insignificant. The same was true for nevus tasks (*r* = 0.130, *p* = 0.249). However, there was a significant negative correlation between the number of solved tasks and the task performance in melanoma tasks (*r* = − 0.272, *p* = 0.015).

Figure 4 suggests that there might be a relation between the overall task performance and the number of solved tasks in the first 50 tasks. Hence, we also calculated Pearson correlations only for this section. Indeed, when only considering the first 50 tasks, there was a significant positive correlation between the number of solved tasks and the overall task performance (*r* = 0.369, *p* = 0.008) and the nevus task performance (*r* = 0.429, *p* = 0.002). However, the correlation between the number of solved tasks and the melanoma task performance was not significant when only considering the first 50 tasks (*r* = − 0.106, *p* = 0.465).  We provide some further analyses regarding the number of solved tasks in Appendix E.

 (3) Response time

From an *inter-tasks perspective*, we did not find a significant Pearson correlation between overall task performance and task time (*r* = 0.017, *p* = 0.788). Separated analyses showed that for the melanoma tasks, the correlation between task performance and task time was also non-significant (*r* = − 0.140, *p* = 0.127). However, for the nevus task, we found a significant positive correlation between task performance and task time (*r* = 0.182, *p* = 0.047).

Furthermore, from an *intra-tasks perspective*, the paired samples *t*-test for all tasks revealed no significant difference between the task times for incorrect (*M* = 3.11 s, *SD* = 0.73 s) versus correctly (*M* = 3.12 s, *SD* = 0.54 s) solved tasks (*t*(239) = 0.116, *p* = 0.908, Cohen’s *d* = 0.007). When considering only the melanoma tasks, however, the task time for false(-negative) diagnoses was significantly higher (*M* = 3.29 s, *SD* = 0.88 s) than the task time for true(-positive) diagnoses (*M* = 3.04 s, *SD* = 0.54 s; *t*(119) = 2.390, *p* = 0.009, Cohen’s *d* = 0.218). On the contrary, when considering only the nevus tasks, the task time for false(-positive) diagnoses was significantly lower (*M* = 2.93 s, *SD* = 0.48 s) than for true(-negative) diagnoses (*M* = 3.20 s, *SD* = 0.55 s; *t*(119) = -3.469, *p* < 0.001, Cohen’s *d* = 0.317).

From an *inter-individuals perspective*, the participants’ accuracy was significantly positively correlated with their individual time (*r* = 0.277, *p* = 0.001).

**Figure 5:**
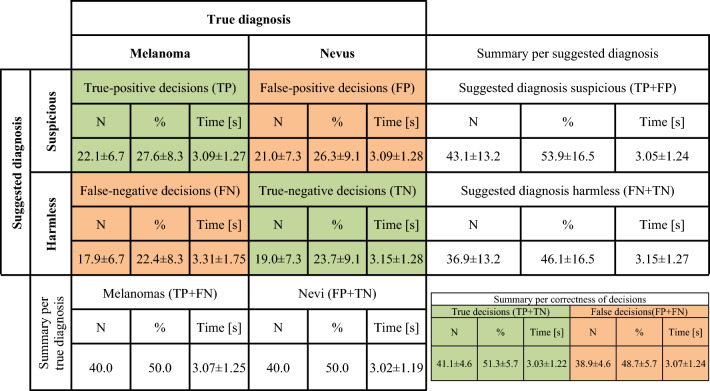
Confusion matrix of an average participant. *Legend:* N = Number of tasks, % = Proportion of tasks, Time = Individual response time in seconds (average based on median per participant); Values display means ± standard deviations based on 137 participants and 80 skin lesion classification tasks per participant.

From an *intra-individuals perspective*, the paired samples *t*-tests to compare the individual times for different types of false versus true decisions were only significant in melanoma tasks (FN: *M* = 3.31 s, *SD* = 1.75 versus TP: *M* = 3.09 s, *SD* = 1.27 s; *t*(136) = 1.871, *p* = 0.032, Cohen’s *d* = 0.160) and when the suggested diagnosis was *harmless* (FN: *M* = 3.31 s, *SD* = 1.75 s versus TN: *M* = 3.15 s, *SD* = 1.28 s; *t*(136) = 1.668, *p* = 0.049, Cohen’s *d* = 0.143). Figure 5 shows the confusion matrix of an average participant in the overview. ﻿Appendix F contains selected additional analyses regarding the response time.

## Discussion

### Summary and discussion of results

In this explorative study, we investigated three factors related to laypersons’ performance in diagnosing pigmented skin cancer: the characteristics of the skin lesions, the number of solved tasks, and the response time.

In general, the average proportion of 51.3% correct answers was only slightly better than a random decision, confirming that, on average, laypeople could not classify skin lesions correctly. However, the large standard deviation of 20.7% and the range of 8.89% to 95.7% showed that considerable differences exist between the images. Fewer than 50% of the participants solved some of the classification tasks correctly. This indicates that some lesions are not only difficult to classify in a classical sense but somewhat misleading. Still, in the case of medical image classification, it is essential to include these tricky cases in learning interventions and performance assessments because they are a likely reason for misdiagnoses.

Regarding the characteristics of the skin lesions, we analysed whether lesion diagnosis, size and anatomic site, as well as the age and sex of the patients, were related to the task performance. For our sample of laypersons, we found that the task performance was higher for the melanomas versus nevi (*p* = 0.003, Cohen’s *d* = 0.385), which could mean that melanomas of this dataset were less challenging to classify. However, an alternative explanation is that the participants generally tended to classify lesions as suspicious (Appendix D).

A further investigation of the melanoma tasks separately did not reveal any significant relations between the task performance and the clinical size and the anatomic site of the lesion, as well as the age and sex of the patient. Similarly, a closer inspection of the nevus tasks also did not reveal significant findings for the clinical size of the lesion as well as the age of the patient. However, we discovered that the nevi from female patients were classified correctly more often than the nevi from male patients (*p* = 0.021, Cohen’s *d* = 0.377). Still, this finding could also have come about due to chance and should be confirmed in a replication study before further interpretation.

Regarding the number of solved tasks, we found that the proportion of participants who provided the correct answer slightly increased over the first 50 tasks, especially for nevi, notably without any feedback. As the performance dropped after about task 50, this could indicate that a threshold for becoming tired or bored was reached.

Regarding the response time, we did not find a significant correlation with the performance in melanoma tasks. In contrast, for nevus tasks, the performance was higher when the response time was longer. A potential explanation for this finding is that the participants looked for *suspicious* features and decided as soon as they found them, leading to longer response times for *true* decisions in nevi.

Furthermore, in line with previous research^17^, we found longer response times for false versus true decisions, but only in melanoma tasks and when the suggested diagnosis was *harmless* and only with small—probably clinically irrelevant—effect sizes of Cohen’s *d* = 0.160 and 0.143, respectively^22^. However, these effect sizes should be contextually interpreted^23^, and it should be considered that, especially for the within-individuals response time comparisons, even minor effects can be meaningful. That said, it is striking that for both significant false versus true comparisons, the comparison is made with the time spent on false-negative decisions, which is consistent with the previously mentioned suggestion that the participants spent time looking for signs of suspicion. Furthermore, this suggests that, when using response time as a marker for confidence, it might be advisable to use the individual means per each of the four outcomes (TP, TN, FP, and FN) separately instead of the overall individual mean^20^.

### Limitations and strengths

A significant limitation of our findings is that we conducted the study with Amazon Mechanical Turk participants^24,25^, who were motivated by a performance-based financial incentive. While we intended to nudge participants towards giving their best with this form of compensation, empirical evidence suggests that payment has surprisingly little effect on cognitive training outcomes^26^. Nevertheless, we carefully analysed the participants’ answering patterns and excluded them from data analysis if they showed suspicious behaviour, for example, always suggesting the same diagnosis. Still, our results are only informative for laypersons. Future research should, therefore, check whether our findings can be replicated with non-M-Turk novices in skin lesion classification and compare the results with insights on experts’ behaviour. Finally, our work only covers the distinction between melanomas and nevi, even though other types of prevalent skin lesions, such as seborrheic keratoses, also show considerable resemblance. Thus, it would be advisable for future investigations to extend the image dataset to additional skin conditions.

As a major strength of our study, we would like to highlight that we used strict inclusion criteria for the images we used to create the skin lesion classification tasks. These criteria guaranteed that the two types of skin lesions were challenging to distinguish, which is confirmed by our empirical results. Nevertheless, the dataset encompassed images from diverse sources (which means multiple dermatoscopes handled by different dermatologists), which could notably have influenced the image quality and, thus, the classification outcomes. Another strength of our research is the rigorous and comprehensive analysis of the findings from multiple perspectives, which allowed us to gain novel, in-depth insights into the interaction of laypeople with skin lesion classification tasks.

## Conclusions

This explorative study showed how the characteristics of the skin lesions, the number of solved tasks, and the response time are associated with the performance of laypersons diagnosing pigmented skin cancer. The generated knowledge can inform the design and evaluation of learning interventions for melanoma detection. Concretely, our empirically assessed task performance values can be used to indicate task difficulty, as suggested by classical test theory^27^. This enables researchers and educators to make informed decisions about the intended difficulty of diagnostic tests and learning interventions for melanoma detection. Illustratively, the findings of two recently published studies underline the importance of conscious design of learning interventions and stratified analyses for easy, medium and difficult classification tasks^10,11^. Furthermore, the current findings showed that researchers can expect small performance increases when individuals have to solve multiple subsequent visual diagnostic tasks. However, they should also be aware of potential trade-offs with fatigue effects after approximately 50 tasks. Finally, our in-depth analysis of response times indicated that it might be advisable for future studies to look at true-positive, true-negative, false-positive, and false-negative decisions separately rather than just associating response times with true or false outcomes, respectively. Researchers can use the previously existing image materials from the ISIC archive^21^ and combine them with the information about the performance-related factors we provide in the datasets published together with this article to design learning activities or diagnostic tests, which can finally contribute towards the improvement of teaching melanoma detection.

### Supplementary Information

Supplementary Information.

## Data Availability

The datasets generated and analysed during the current study are available in the Open Science Framework repository, https://osf.io/aundt/?view_only=8fb61dce0c2f4efdbd5fe08410bbbf04.

## Abbreviations

TP: True-positive
TN: True-negative
FP: False-positive
FN: False-negative
