# Supplementary material for: Factors related to the performance of laypersons diagnosing pigmented skin cancer: an explorative study
Source: Sci Rep. 2023 Dec 21;13:22790. doi: 10.1038/s41598-023-50152-x (PMC10733329; doi:10.1038/s41598-023-50152-x)
Supplement: Supplementary file 1 — Supplementary Information. [file 41598_2023_50152_MOESM1_ESM.pdf]

Factors related to the performance of laypersons diagnosing pigmented skin cancer: an explorative study – Appendices

Nadja Beeler, Esther Ziegler, Alexander A. Navarini and Manu Kapur

Contents

Appendix A. Details on outlier removal 2

Appendix B. Inclusion criteria and characteristics of skin lesion images 3

Appendix C. Post-randomisation checks for distribution of images to surveys 5

Appendix D. Additional analyses – 1) Characteristics of skin lesions 7

Appendix E. Additional analyses – 2) Number of solved tasks 10

Appendix F. Additional analyses – 3) Response time..12

References 14

Appendix A. Details on outlier removal

To exclude people who presumably did not take the study seriously or who were distracted during their participation, we removed eight participants who were below the 1st quartile – 1.5*interquartile range or above the 3rd quartile + 1.5*interquartile range for the total time that they needed for completing all 80 skin lesion classification tasks. Additionally, we excluded four participants who spent, on average, less than 1.5 seconds per task. Finally, we excluded five individuals whose answers contained obvious patterns, for example, always providing the same answer.

Appendix B. Inclusion criteria and characteristics of skin lesion images

The main inclusion criteria for the skin lesion images are displayed in Table S1. 413 nevus and 145 melanoma images in the ISIC archive fulfilled these criteria [1]. For the final selection, we applied additional, subjectively rated secondary criteria, also shown in Table S1. Finally, Table S2 provides an overview of the characteristics of the selected images.

Table S1: Inclusion criteria for the investigated images

**Main criteria**	
Dataset	MSK-2 [2]
Lesion diagnosis	Melanoma (malignant) or nevus (benign)
Type of diagnosis	Histopathology
Image type	Dermoscopic
General anatomic site of the lesion	Torso (anterior/lateral/posterior),<br>Extremities (lower/upper), or<br>Head/neck<br>(Excluded: Palms/soles)
Clinical size – longest diameter of the lesion	0-10 mm
Approximate age of the patients	40-80 years
**Secondary criteria**	
Image quality	Good quality (clear picture)
Amount of hair	Not too much hair, skin lesion well visible
Marker	No marker on the picture
Bubbles	No, or not too many bubbles between the lens of the camera and the skin of the patient

Table S2: Characteristics of the selected images

	Images of melanomas	Images of nevi	Comparison of melanomas vs. nevi
**Clinical size of the lesions**	Longest diameter (mm)		Student's t-test for independent samples
	M = 5.8, SD = 1.9	M = 4.9, SD =1.8	$t(238) = 3.955$, $p < 0.001$, Cohen's $d = 0.511$
**Age of the patients**	Age (years)		Student's t-test for independent samples
	M = 60, SD = 10.5	M = 52.8, SD =9.5	$t(238) = 5.634$, $p < 0.001$, Cohen's $d = 0.727$
**Anatomical site of the lesions**	Number of images (per cent images per category in brackets)		Pearson Chi-Squared test
Torso	45 (37.5%)	66 (55.0%)	X^2 (2, N = 240) = 18.219, $p < 0.001$
Extremities	47 (39.2%)	48 (40.0%)	
Head/neck	28 (23.3%)	6 (5.0%)	
Total	*120 (100%)*	*120 (100%)*	
**Sex of the patients**	Number of images (per cent images per category in brackets)		Pearson Chi-Squared test
Female	51 (42.5%)	65 (54.2%)	X^2 (1, N = 240) = 3.271, $p = 0.071$
Male	69 (57.5%)	55 (45.8%)	
Total	*120 (50%)*	*120 (50%)*	

Appendix C. Post-randomisation checks for distribution of images to surveys

The results of the post-randomisation checks to see how the characteristics of the skin lesion images are distributed to the three surveys are displayed in Table S3, Table S4, Table S5 and Table S6.

Clinical size of the lesions

Table S3: Clinical size of the lesions (longest diameter), per survey

	Size of the lesions (in mm)		
	Mean	*SD*	*N*
Survey 1	5.5	1.9	80
Survey 2	5.2	1.8	80
Survey 3	5.3	1.9	80

F (2, 237) = 0.149, p = 0.862

Age of the patients

Table S4: Age of the patients, per survey

	Age of the patients (in years)		
	Mean	*SD*	*N*
Survey 1	56.8	10.8	80
Survey 2	56.6	10.1	80
Survey 3	55.9	11.1	80

F (2, 237) = 0.641, p = 0.528

Anatomical site of the lesions

Table S5: Anatomic sites of the lesions, per survey

	Anatomic site of the lesions			
	Torso	Head/neck	Extremities	Total
Survey 1	36 (45.0%)	11 (13.8%)	33 (41.3%)	80 (100.0%)
Survey 2	40 (50.0%)	10 (12.5%)	30 (37.5%)	80 (100.0%)
Survey 3	35 (43.8%)	13 (16.3%)	32 (40.0%)	80 (100.0%)

Pearson Chi-square (4) = 0.938, p = 0.919

Sex of the patients

Table S6: Sex of the patients from which the lesions were excised, per survey

	Sex of the patients		
	Female	Male	Total
Survey 1	41 (51.2%)	39 (48.8%)	80 (100%)
Survey 2	37 (46.3%)	43 (53.8%)	80 (100%)
Survey 3	38 (47.5%	42 (52.5%)	80 (100%)

Pearson Chi-square (2) = 0.434, p = 0.805

Appendix D. Additional analyses – 1) Characteristics of skin lesions

Comparison of proportions of suggested diagnoses

We ran additional analyses to check whether participants' general tendencies towards suggesting a specific diagnosis might have influenced the observed difference in the task performance for melanomas versus nevi. In our binary decision tasks, this means that if participants generally tended towards diagnosing a lesion as *suspicious* (or *harmless*) when uncertain, they would be more likely to classify more melanomas (or nevi) correctly. Hence, we compared the proportions of all suggested diagnoses (*suspicious* or *harmless*).

To do this, we used a one-sample *t*-test to compare the proportion of the suggested diagnosis *suspicious* with a ratio of 50% (which would be expected if participants equally suggested the two diagnoses). If this proportion is significantly different from 50%, then this is also the case for the proportion of the suggested diagnosis *harmless* because these proportions sum up to 100%.

The one-sample *t*-test showed that the proportion of the suggested diagnosis *suspicious* was significantly higher (M = 53.9%, SD = 20.3%) than a to-be-expected proportion of 50% ($t(239) = 40.680$, $p < 0.001$, Cohen's $d = 2.626$). This indicates that the higher task performance for melanoma tasks might partly be explained by a general tendency of the participants to suggest a *suspicious* lesion. Still, it could also be the case that the proportion of suggested *suspicious* lesions is higher because the participants are better at classifying melanoma (i.e., if they would classify all melanoma correctly, they would most likely also have a higher proportion of suggested *suspicious* lesions).

Potential explanations for higher task performance in nevi from female patients

We found that the task performance was higher in nevi stemming from female versus male patients, so we conducted further tests to explore potential explanations.

First, we conducted an additional one-sample *t*-test to check whether the result might partly be due to a general tendency towards suggesting that the lesions are *harmless* (which is the correct diagnosis in nevus tasks) when they stem from female patients. However, the opposite was the case: The proportion of suggested diagnosis *harmless* for all lesions from female patients was significantly *lower* (M = 47.1%, SD = 20.4%) than an expected proportion of 50% ($t(115) = 24.561$, $p < 0.001$, Cohen's $d = 2.280$).

Second, another one-sample *t*-test showed that the proportion of suggested diagnosis *harmless* was also lower when testing separately for the lesions stemming from male participants (M = 45.1%, SD = 20.3%, t(123) = 24.495, p < 0.001, Cohen's d = 2.200). This means that the participants had a significant tendency towards *not* suggesting a *harmless* lesion irrespective of whether the lesions stemmed from female or male patients.

Third, an independent samples *t*-test revealed that the difference between the proportions of suggested *harmless* diagnoses in lesions stemming from female or male patients was insignificant ($t(238) = 0.752$, $p = 0.453$, Cohen's $d = 0.097$). This means that there was no significant difference in the extent of the tendency not to suggest a *harmless* lesion between lesions stemming from female versus male patients.

These results of the additional analyses indicate that the higher task performance for nevus lesions stemming from female versus male patients cannot be explained by a general tendency towards suggesting a *harmless* lesion in lesions stemming from a female patient. As an alternative explanation, we analysed whether there were differences in the characteristics of the nevi from female vs. male patients.

However, *t*-tests for independent samples revealed no significant differences neither in the size of the nevi stemming from females (M = 4.8mm, SD = 1.6mm) vs. males (M = 5.0mm, SD = 2.0mm, $t(118) = -0.502$, $p = 0.617$, Cohen's d = -0.092) nor in the age of the female (M = 52.5 y, SD = 9.3 y) vs. male (M = 53.1 y, SD = 9.9 y, $t(118) = -0.359$, $p = 0.360$, Cohen's $d = -0.066$) patients from which the nevi had been excised.

Finally, a Chi-Squared test showed a significant difference in the distribution of the anatomic sites of the nevi from female vs. male patients, X^2 (2, N = 120) = 8.155, p = 0.017). Concretely, 43.1% of the nevi from female patients stemmed from the torso, 50.8% from the extremities and 6.2% from the head/neck. The nevi from male patients stemmed from the torso in 69.1%, the extremities in 27.3%, and the head/neck in 3.6% of the cases. This shows that nevi from female patients stemmed from the extremities more often (and from the torso less often) than nevi from male patients and leads to the assumption that the participants might generally have tended to suggest that lesions are *harmless* in lesions stemming from the extremities versus the torso.

An independent samples *t*-test confirmed that this was the case: The proportion of participants who suggested a *harmless* lesion was significantly higher for (melanoma and nevus) lesions stemming from the extremities (M = 50.74%, SD = 21.0%) versus the torso (M = 42.5%, SD = 19.2%), $t(204)$ = 2.941, p = 0.004, Cohen's d = 0.411). This indicates that lesions from the extremities might generally look more harmless than those from the extremities and vice versa.

Overall, the higher task performance in nevi stemming from female versus male patients can partly be explained by the facts that A) nevi from female patients stemmed from the extremities more often than nevi from male patients, and B) participants generally suggested a harmless lesion more frequently in lesions stemming from the extremities than from the torso.

Appendix E. Additional analyses – 2) Number of solved tasks

Suggested diagnosis and number of solved tasks

To check whether a potential relationship between the task performance and the number of solved tasks could (partly) be explained by the proportion of suggested diagnoses (see also Appendix D), we plotted the moving average of the proportion of suggested *suspicious* lesions over the number of solved tasks. Furthermore, we calculated Pearson correlations between the number of solved tasks and the proportion of suggested diagnoses *suspicious* (from which one can calculate the proportion of suggested diagnoses *harmless*: % *harmless* = 100% - % *suspicious*).

Figure S1 displays that for the first few tasks, the proportion of the participants who suggested the diagnosis *suspicious* was more than 50%. However, starting from about task 10, this proportion stays relatively stable, slightly above 50%.

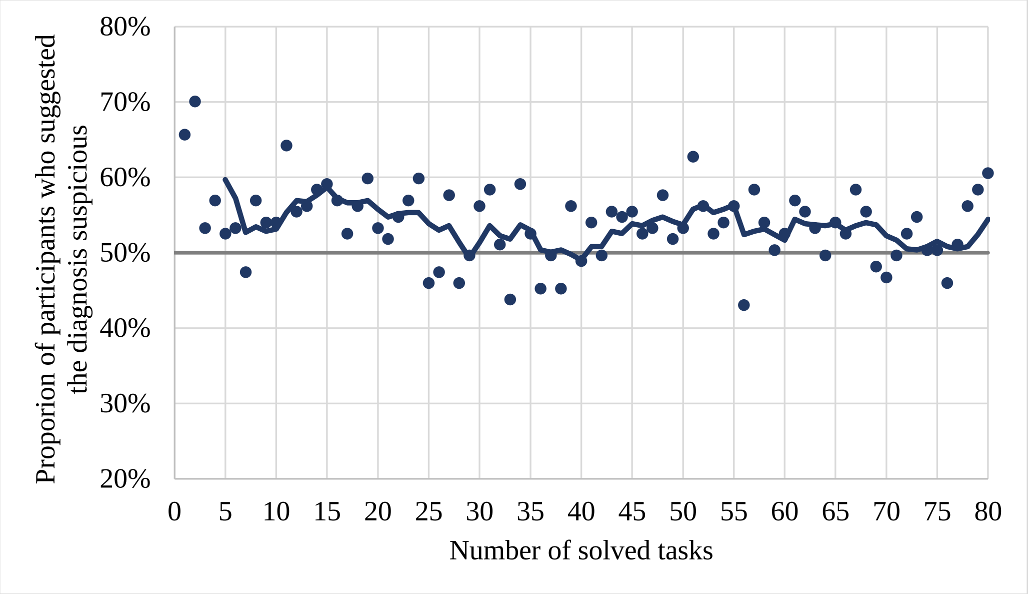

Figure S1: Proportion of participants who suggested a *suspicious* lesion over the number of solved tasks
Note: Dots represent the proportion in single tasks; lines represent the moving average of the proportion over five tasks. An average proportion of 50% could be expected by chance (grey line).

Furthermore, there was a significant negative correlation between the number of solved tasks and the proportion of the suggested diagnosis *suspicious* (r = -0.239, p = 0.032). Because the proportions of the suspected diagnoses, *suspicious* and *harmless*, sum up to 100%, this can be interpreted as a positive correlation between the number of solved tasks and the proportion of the suggested diagnosis *harmless*. The negative correlation between the number of solved tasks and the suggested diagnosis suspicious persisted when only considering the first 50 tasks (r = -0.367, p = 0.009).

Appendix F. Additional analyses – 3) Response time

Intra-tasks perspective

To compare the task times between the two answer options, we conducted a dependent samples *t*-test. It revealed that based on all tasks, the task time was significantly longer for the suggested diagnosis *harmless* (M = 3.24 s, SD = 0.73 s) than for the suggested diagnosis *suspicious* (M = 2.99 s, SD = 0.51 s; $t(239) = 3.985$, $p < 0.001$, Cohen's d =0.257).

Inter-individuals perspective

To assess the relationship between individual performance and individual response time for melanoma and nevus tasks separately, we first calculated the participant performance in melanoma tasks (sensitivity = true-positive rate = TP/(TP+FN)) and nevus tasks (specificity = true-negative rate = TN/(TN+FP)). Subsequently, we calculated Pearson correlations between sensitivity and individual time and between specificity and individual time.

We found a significant positive correlation between the specificity and the individual time ($r = 0.254$, $p = 0.003$). However, we found no significant correlation between the sensitivity and the individual time ($r = -0.087$, $p = 0.312$).

Intra-individuals perspective

To complete the picture from an intra-individuals perspective, we conducted two-sided paired samples *t*-tests to compare the individual response times in melanoma versus nevus tasks (TP & FN vs. FP & TN) and for the suggested diagnosis *harmless* versus *suspicious* (TN & FN vs. TP & FP).

We found that the individual response time was higher in melanoma tasks (TP & FN: M = 3.07 s, SD = 1.25 s) compared to nevus tasks (FP & TN: M = 3.02 s, SD = 1.19 s; $t(136)$

= 2.136, $p = 0.034$, Cohen's $d = 0.182$). The difference in the individual response times for suggesting the diagnosis *harmless* (TN & FN: $M = 3.15$ s, SD =1.27 s) versus *suspicious* (TP & FP: $M = 3.05$ s, $SD = 1.24$ s) was non-significant ($t(136) = 1.841$, $p = 0.068$, Cohen's d = 0.157).

References

[1] International Skin Imaging Collaboration: Melanoma Project, ‘ISIC Archive’, *Gallery*. https://www.isic-archive.com (accessed Nov. 06, 2020).

[2] N. C. F. Codella *et al.*, ‘Skin Lesion Analysis Toward Melanoma Detection: A Challenge at the 2017 International Symposium on Biomedical Imaging (ISBI), Hosted by the International Skin Imaging Collaboration (ISIC)’, *arXiv:1710.05006 [cs]*, Jan. 2018, Accessed: Nov. 06, 2020. [Online]. Available: http://arxiv.org/abs/1710.05006
